# FOXM1 in sarcoma: role in cell cycle, pluripotency genes and stem cell pathways

**DOI:** 10.18632/oncotarget.8669

**Published:** 2016-04-09

**Authors:** Fergal C. Kelleher, Hazel O'sullivan

**Affiliations:** ^1^ St. James Hospital, Dublin, Ireland; ^2^ Trinity College Dublin, Dublin, Ireland; ^3^ Whangarei Base Hospital, Whangarei, New Zealand

**Keywords:** FOXM1, pluripotency genes, thiazole antibiotics, DREAM, mesenchyme

## Abstract

FOXM1 is a pro-proliferative transcription factor that promotes cell cycle progression at the G1-S, and G2-M transitions. It is activated by phosphorylation usually mediated by successive cyclin – cyclin dependent kinase complexes, and is highly expressed in sarcoma. p53 down regulates FOXM1 and FOXM1 inhibition is also partly dependent on Rb and p21. Abnormalities of p53 or Rb are frequent in sporadic sarcomas with bone or soft tissue sarcoma, accounting for 36% of index cancers in the high penetrance *TP53* germline disorder, Li-Fraumeni syndrome.

FOXM1 stimulates transcription of pluripotency related genes including SOX2, KLF4, OCT4, and NANOG many of which are important in sarcoma, a disorder of mesenchymal stem cell/ partially committed progenitor cells. In a selected specific, SOX2 is uniformly expressed in synovial sarcoma. Embryonic pathways preferentially used in stem cell such as Hippo, Hedgehog, and Wnt dominate in FOXM1 stoichiometry to alter rates of FOXM1 production or degradation. In undifferentiated pleomorphic sarcoma, liposarcoma, and fibrosarcoma, dysregulation of the Hippo pathway increases expression of the effector co-transcriptional activator Yes-Associated Protein (YAP). A complex involving YAP and the transcription factor TEAD elevates FOXM1 in these sarcoma subtypes. In another scenario 80% of desmoid tumors have nuclear localization of β-catenin, the Wnt pathway effector molecule. Thiazole antibiotics inhibit FOXM1 and because they have an auto-regulator loop FOXM1 expression is also inhibited. Current systemic treatment of sarcoma is of limited efficacy and inhibiting FOXM1 represents a potential new strategy.

## INTRODUCTION

Soft tissue sarcomas are heterogeneous mesenchymal tumors. The American Cancer Society in 2015 documented an incidence rate of 11,930, with an anticipated annual death rate of 4,870. Treatment of metastatic disease usually comprises doxorubicin and ifosfamide either alone or in combination, with a greater than 40% rate of 12- week progression free survival considered a descriptor of second-line treatment efficacy. Therefore the benefit from systemic treatment of this disease is modest with a need for new therapeutics. One candidate strategy is targeted inhibition of the mammalian transcription factor Forkhead Box M1 (FOXM1).

## FOXM1 AND THE CELL CYCLE

FOXM1 is a member of the Forkhead transcription factor family. Constituent members have an evolutionarily conserved, 100-amino acid long DNA binding domain called Forkhead or ‘winged helix’ [1, 2]. In *Drosophila melanogaster* mutations of forkhead create ectopic head structures in the fruit fly embryos, hence the nomenclature. There are 19 different subgroups, FOX1-FOXS, categorized on the basis of sequence homology inside and outside the forkhead domain. In particular FOXA, FOXC, FOXM, FOX0 and FOXP are essential components of oncogenic and tumor suppressive pathways. FOXM1 is a crucial pro-proliferative transcription factor, which is activated by phosphorylation. It also has an upregulating auto regulatory loop [3]. It is induced by oncoproteins such as MYC and KRAS and repressed by products of tumor suppressor genes such as CHK2 and TP53 [4–6]. FOXM1 transcriptionally activates important pro-proliferative genes and promotes cell cycle progression at the G1/S and G2/M transitions. The cyclin-dependent kinases CDK4/6 phosphorylate FOXM1 to facilitate continued expression of G1/S phase genes [7]. FOXM1 undergoes cytoplasmic accumulation in late G1 and S phases, followed by cyclin E-CDK2 / Raf-MEK-ERK mediated phosphorylation, nuclear translocation and entry into G2-M phase [8, 9]. In normal cells, FOXM1 is phosphorylated in the S to G2 phases, and undergoes ubiquitin dependent proteasomal destruction during the M to G1 phase.

Cyclin/CDK complexes mediate cell cycle progression with their effects partly executed by altering transcription factors such as FOXM1 or E2F. The E2F1 transcription factor also contributes to the expression of FOXM1 [1]. Cyclins markedly activate the catalytic activity of their serine/threonine cyclin dependent kinase partner with activity of FOXM1 mediated by successive phosphorylation events. RB is also an important substrate for cyclin-CDK complexes. Early in the cell cycle at M/G1 transition almost all of the phosphate groups are removed from retinoblastoma protein (pRb) resulting in an unphosphorylated configuration. With progression through the G1 phase a single phosphate group is attached to any of 14 potential phosphorylation sites. At the restriction point in late G1 phase, pRb is phosphorylated by cyclin E- CDK2 complexes at a minimum of 12 more sites creating a hyperphosphorylated state, which persists until entry into the M phase. The active form of RB is the unphosphorylated protein, which binds cellular proteins including E2F. E2F family transcription factors are required for expression of S-phase genes. When pRb is hyperphosphorylated this causes the release of transcription factors including E2F permitting G1 to S phase transitions and cell cycle progression [10, 11]. Importantly two potential E2F binding sites have been identified in the FOXM1 promoter [1]. The FOXM1 promoter also binds B-Myb and CHR-NF-Y. Children with hereditary retinoblastoma, a condition in which tumors arise from biallelic functional loss of *RB1*, have a 1,000 fold increased risk of osteosarcoma compared to the general population. Furthermore *RB1* alterations are identified in 80% of primary sporadic osteosarcomas [12–14]. Amplification of *CDK4* and loss of *RB1*, or *CDKN2A* loss are considered nearly universal in osteosarcoma with 20% of cases having either amplification of *CDK4* or deletion of *CDKN2A* [15]. These alterations lead to G1/S deregulation.

Growth factors neutralize the inhibitory effects of Rb by its successive phosphorylation. The G1/S checkpoint is the first important checkpoint in the cell cycle and it involves both the RB and p53 proteins. RB and p53 have been implicated in numerous sarcoma subtypes. Mice with osteoblast-restricted deletion of p53 and pRb develop short latency high-grade osteosarcoma [11]. In childhood survivors of retinoblastoma, osteosarcoma is the most common subsequent malignancy to arise, itself a disease stemming from homozygous functional loss of *RB*. In this population osteosarcoma can originate either in irradiated or non-irradiated tissue and represents 25%-40% of second cancers [16, 17]. The DREAM (Dimerization partner, RB-like, E2F and multi-vulval class B (MuvB)) complex mediates gene repression in G0, early and late phases of the cell cycle and co-ordinates periodic gene expression. Peaks in gene expression occur in G1/S and G2/M [18]. E2F is required for G1/S phase gene expression. MuvB core complex, BMYB and FOXM1 are required for G2/M phase gene expression. BMYB (a transcription factor phosphoprotein) and FOXM1 are phosphorylated and undergo proteasomal destruction during respective S to G2, and M to G1 phases. In cancer increased BMYB-MuvB-FOXM1 activity supervenes with loss of DREAM function with a shift in cellular balance from quiescence to proliferation.

The p53 transcription factor is a tumor suppressor protein, which down-regulates FoxM1. *TP53* is mutated in numerous cancers including many sarcoma subtypes. The highly penetrant cancer predisposition disorder Li-Fraumeni syndrome is associated with germline TP53 mutations. The index cancers in 36% of patients with Li-Fraumeni syndrome are either bone or soft tissue sarcomas [19]. *TP53* mutations occur in 19% to 38% of sporadic osteosarcomas [20, 21].

**Figure 1:**
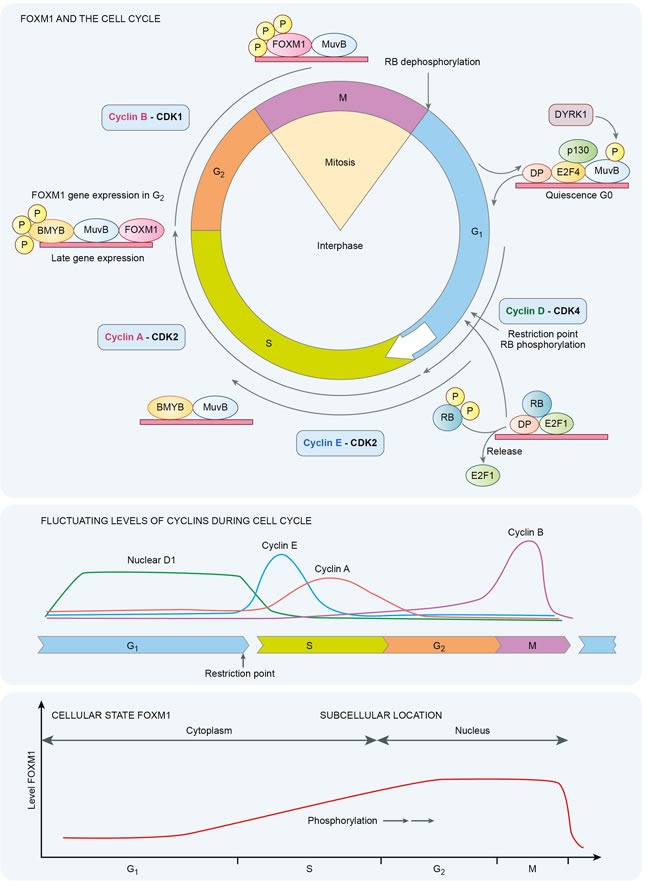
Temporal dynamics of FOXM1 in the cell cycle

FOXM1 is itself a transcription factor that regulates numerous G2/M specific genes. Inhibition of FOXM1 is also partly dependent on Rb and p21. Secondary to DNA damage, p53 can participate in repressing FoxM1 mRNA and thus FOXM1 protein expression. A global gene expression analysis of different cancer types found FOXM1, E2F1, and MYBL2 are disproportionately upregulated in p53 mutant cancers [22]. MYB-related protein B (B-MYB/MYBL2) coordinates cell cycle transition in conjunction with multivulval class B (MuvB) complex and FOXM1[23]. The binding of FoxM1 to the promoters of genes involved in G2/M is dependent on the transcription factor B-Myb [24]. The transcriptional activation of FoxM1 requires sequential phosphorylation by cyclin dependent kinases and PLK1. These are target genes of B-Myb. Therefore B-Myb enables FoxM1 binding to G2/M gene promoters. Microarray analysis of breast cancers demonstrates that FOXM1 regulates genes essential for chromosomal segregation and mitosis with loss of FOXM1 leading to mitotic spindle defects and mitotic catastrophe [25]. FOXM1 is progressively phosphorylated with initiation of phosphorylation in mitosis as demonstrated in Figure 1, and is involved in G2-M phases of the cell cycle. Expression of FOXM1 is increased during G1 and S phases and is maximal in G2-M phases of the cell cycle with overexpression of FOXM1 stimulating expression of cyclin B1[26].

## FOXM1 AND MESENCHYMAL TUMORS

FOXM1 has pleiotropic roles in embryogenesis in which it is almost ubiquitously expressed, particularly in proliferating mesenchymal and epithelial cells. In adult tissues expression is restricted to actively dividing cells of selected organs with expression eliminated in quiescent or terminally differentiated cells [2]. Interestingly the Rb pathway controls both quiescence and senescence. A senescence - quiescence switch mediated by cross talk between the Rb-AKT signaling pathways, FoxO3a and FoxM1 transcription factors has been identified in cultured fibroblasts [27]. Senescence is an important barrier to cancer, which is overcome with development of malignancy. Furthermore in these studies E2F1 and E2F3 were found to bind to and activate the gene promoter of *FOXM1* in proliferating fibroblasts. In senescent cells, CDK4 and CDK6, targets of p16^INK4a^ stabilize FOXM1 protein [7]. A systematic screen of CDK4/6 substrates identified phosphorylation of FOXM1 as a common target [7]. CDK4/6 stabilized and activated FOXM1, thereby maintaining G1/S phase gene expression and suppressing reactive oxygen species levels. Phosphorylation of FOXM1 suppressed senescence in malignant cells. In malignant peripheral nerve sheath tumors, amplification of the *CDK4* gene and increased levels of FOXM1 protein are significant independent predictors of poor survival [28].

The World Health Organization has a tissue of origin classification system for sarcomas with subtype assignment determined by histiogenesis. Limitation arises as it is not possible to assign a lineage of origin to some poorly differentiated tumors. Interestingly in two malignancies separate from sarcoma, embryonic carcinoma and neuroblastoma, a role for FOXM1 has been identified in the maintenance of the undifferentiated tumor state of the malignant cells. This arises by FOXM1 activating pluripotency-associated genes [29, 30]. Sarcoma likely arises from stem cells or partly committed progenitor cells. A suggested inference is that FOXM1 has a similar role in undifferentiated pleomorphic sarcoma. It is a diagnosis of exclusion as these tumors defy lineage assignment. This postulate is partly substantiated by recent experimental evidence in which deregulated Hippo pathway signaling in soft-tissue sarcomas was found to promote FOXM1 expression and tumorigenesis in undifferentiated pleomorphic sarcoma, fibrosarcoma and liposarcoma [31]. Most soft-tissue sarcomas arise from tissues derived from the mesodermal or ectodermal germ layers, however some such as angiosarcomas have an endodermal origin. Therefore the ontological merit in evaluating FOXM1 as a therapeutic target in sarcoma, especially undifferentiated pleomorphic sarcoma, is apparent. Furthermore FOXM1 participates in epithelial to mesenchymal transition, a determinant of the morphological subtypes of synovial sarcoma.

FOXM1 levels can be increased by gene amplification and translocation, or by altered kinetics of production and degradation. *FOXM1* maps to chromosomal band 12p13 [32]. This locus is amplified in several types of cancer with *FOXM1* amplification among the most prevalent molecular aberration in non-Hodgkin's lymphoma [33]. Chromosome band 12q13 is amplified in malignant peripheral nerve sheath tumors [34]. Comparative genomic hybridization of these tumors has permitted correlation of regional copy number gains with decreased survival [35, 36]. *FOXM1* emerged as one candidate amplified gene, however on multivariate analysis *CDK4,* also on chromosome 12q13, was the most significant forecaster of poor survival [28]. Amplification of chromosomal region 12q13-15 is also found in well-differentiated liposarcomas within supernumerary ring and giant marker chromosomes. The proto-oncogenes *CDK4* and *MDM2* are both amplified in well-differentiated liposarcoma. Translocations involving chromosome band12q13 occur in other soft tissue sarcomas subtypes. Myxoid/round cell liposarcoma has a translocation t (12q13; 16p11) that creates a *FUS*-*DDIT3* gene fusion. Clear cell sarcoma has a translocation t (12q13; 22q12) that creates an *EWSR1*-*WT1* gene fusion. Infantile fibrosarcoma has a translocation t (12q13; 15q26) that creates an *ETV6*-*NTRK3* gene fusion. The frequency of the described fusion genes in the respective malignancies is each ~75%. Considering a phylogenetic map of soft tissue sarcomas (which comprises ~65 histological types), Ewing sarcoma family tumors and clear cell sarcoma occupy a common second order phylogenetic branch. FOXM1 is of established importance in both these malignancies. Well-differentiated liposarcomas and myxoid liposarcomas arise from a common first order branch but are separate from other FOXM1 candidate soft tissue sarcomas.

Direct binding to the FOXM1 promoter by E2F or c-Myc increases its expression by up regulating gene transcription. Conversely, p53 negatively regulates expression of FOXM1[37]. Mutation or inactivation of p53 frequently occurs in malignancies and may increase FOXM1 expression. Mice with p53 deficiency develop spontaneous sarcomas and thymic lymphoma [38, 39]. Li-Fraumeni syndrome, which arises from germline mutations in *TP53*, is informative. Affected individuals are at markedly increased risk of developing the following six core malignancies: CNS tumors, breast cancer, acute leukemia, adrenocortical carcinoma, soft tissue and bone sarcomas. [40]. Loss of function alterations in p53 typically leads to resistance to apoptosis because p53 stimulates pro-apoptotic gene expression including Noxa, Bad, Bax, DR4, Puma, Apaf1 and caspase-6 [41]. P53 loss is a frequent molecular aberration in sporadic sarcoma.

The FOXM1c isoform binds to and transactivates the E-cadherin gene promoter at a conserved FOXM1 binding site in mice and humans [42]. E-cadherin is a trans-membrane glycoprotein with tumor suppressor activity that participates in intercellular adhesion at adherens junctions. Down regulation of E-cadherin is one part of the phenomenon of epithelial-mesenchymal transition (EMT) in which cells lose apical-basal polarity and mesenchymal genes are induced. EMT has diverse biological roles in gastrulation, tumor invasion and metastasis. In synovial sarcoma, a monophasic fibrous variant accounts for ~60% of cases with the remainder being biphasic synovial sarcoma (containing epithelial and spindle cell components), a rare monophasic epithelial type, and a poorly differentiated type. EMT accounts for this variation in histological appearance. The idea that FOXM1 transcriptionally upregulates a tumor suppressor gene is unexpected and a context dependent approach to FOXM1's role in malignancy must be inferred. FOXM1 would appear to be influential in biphasic synovial sarcomagenesis. Contrastingly in non-small cell lung cancer, high expression of FOXM1 is correlated with loss of E-cadherin expression and anomalous immuno-positivity for vimentin [43]. FOXM1 expression is associated with lymph node metastasis and is also an independent adverse predictor of outcome in this variety of lung cancer.

Finally, FOXM1 is important in rhabdomyosarcoma wherein it directly binds to the Bub1b promoter, which has a FOXM1 consensus-binding site [44]. Bub1b is a spindle assembly checkpoint protein that is of critical importance for growth and survival of both alveolar and embryonic rhabdomyosarcoma cells. There is excellent correlation between FOXM1 and Bub1b levels in this disease. A FoxM1/Bub1b signaling pathway is an identified required component for survival and growth of rhabdomyosarcoma [45]. Bub1b knockdown suppresses *in-vivo* tumor growth with regression of established tumors affected by post-mitotic endoreduplication checkpoint mitotic catastrophe. Using a chromatin immunoprecipitation assay Bub1b has been established as a direct transcriptional target of FoxM1. Experimental suppression of FOXM1 transcription using the FOXM1 inhibitor Siomycin (an antibiotic thiazole compound) or shRNA reduced the expression of Bub1b and inhibited cell growth and survival. Interestingly, patients with mosaic variegated aneuploidy syndrome, a rare disorder with has biallelic or heterozygous mutations of the *Bub1B* gene, have constitutional aneuploidy and a predisposition to early childhood cancer including rhabdomyosarcoma, leukemia and Wilm's tumor [44, 46]. Targeting FOXM1 may prove a useful future therapeutic strategy in rhabdomyosarcoma.

## FOXM1 AND EXPRESSION OF PLURIPOTENCY GENE

FOXM1 stimulates transcription of pluripotency-related genes in cancer stem cells, which are cells with properties of multi-lineage differentiation and self-renewal capacity. Experimental evidence substantiating this concept, detailed herein, has been described in neuroblastoma, Ewing sarcoma, embryonal carcinoma and synovial sarcoma. These malignancies are diagnosed in childhood, adolescence and young adults, respectively. Thus, there is a recurring theme of temporal correlation between tumors arising in childhood or early adult life with a pathobiology of upregulated pluripotency genes. Sarcomas of bones are more common in adolescents and young adults. Though as a categorical group soft tissue sarcomas are more common with increasing age many exceptions apply, such as rhabdomyosarcoma- median age at diagnosis 5 years, and synovial sarcoma occurring most often in young adults- average age at diagnosis 35 years [47]. The median age at diagnosis of translocation-associated sarcomas (e.g. desmoplastic small round cell, Ewing, clear cell, synovial, myxoid-round cell liposarcoma) is 20-50 years, and 50-70 years in complex types of sarcoma (e.g. malignant peripheral nerve sheath tumor, fibrosarcoma, leiomyosarcoma, undifferentiated pleomorphic sarcoma).

In neuroblastoma FoxM1 activates SOX2 (Sex determining Y-box 2) expression [48]. SOX2 along with Klf4, Oct4 and c-Myc is one of the four ‘Yamanaka’ transcription factors that reprogram differentiated cells into induced pluripotent stem cells. In Ewing sarcoma an *EWS*-*FLI1* gene translocation is identified in ~85% of cases. This chimeric fusion gene encodes an aberrant transcription factor and is correlated with increased *in vitro* cell line levels of FOXM1 [49]. Raised FOXM1 levels are also expressed in human Ewing sarcoma tumors. There is an established relationship between EWS-FLI1 and the pluripotency genes *SOX2, OCT4*, and *NANOG* in Ewing sarcoma. *EWS-FLI-1* represses the miRNA145 promoter and induces expression of *SOX2* in Ewing sarcoma to initiate the reprogramming of mesenchymal stem cells towards cancer stem cells [50]. In Ewing sarcoma SOX2 enables cell proliferation and survival by regulating p27, p21 and cyclin E to facilitate G1/S phase transition. In a systematic evaluation of Ewing sarcoma cell lines, human samples and xenograft models siRNA mediated inhibition of SOX2 induces apoptosis and G1/S phase arrest [51]. Ewing sarcoma expresses neurogenic genes, and the postulated cell of origin of Ewing sarcoma is either a mesenchymal stem cell or primitive early neuro-ectodermal cell. *EWS-FLI-1* also induces the expression of SOX2, *OCT4* and *NANOG* in human pediatric mesenchymal stem cells but not in the adult counterparts. CD133 is a stem cell marker in Ewing sarcoma and can be used to identify subpopulations of cells with tumor initiating activity as well as the ability to sustain growth when serially xenotransplanted [52]. CD133 expressing Ewing sarcoma cells are capable of chondrogenic, osteogenic and adipocytic differentiation. CD133 expressing cells expressed higher levels of NANOG, SOX2 and OCT4 [53]. Cancer stem cells in Ewing sarcoma are involved in chemo resistance, with *in vitro* musculoskeletal sarcoma stem cells expressing SOX2, OCT3/4 and NANOG [54, 55].

SOX2 is uniformly expressed in synovial sarcoma and its expression is an essential requirement for proliferation of these cells. SOX2 occupancy of the chromatin remodeling complex BAF (SWI/SNF) is correlated with decreased histone H3 lysine 27 trimethylation (H3K27me3) an epigenetic phenomenon. The characteristic t (X: 18)(p11.2; q11.2) translocation of synovial sarcoma fuses the *SS18* gene on chromosome 18 to one of three homologous genes, *SSX1, SSX2* or *SSX4*, which cluster on the X chromosome [56]. SS18 is an integral subunit of the BAF complex and is closely associated with the catalytic Brg subunit. In synovial sarcoma SS18-SSX replaces SSX18 in the BAF complex and evicts the tumor suppressor BAF47 that then undergoes proteasomal degradation [57]. This altered BAF complex binds with the SOX2 locus and reverses polycomb mediated gene repression thereby activating Sox2. A 2-amino acid segment is the decisive discriminator for SNF5 ejection and *SOX2* induction [58] i.e. the non-pathogenic partner gene SSX3 harbors a methionine-isoleucine amino-acid pair, rather than the evicting lysine-arginine amino acid doublet of SSX1, SSX2, and SSX4 within the chimeric SS18: SSX gene fusion. A future therapeutic approach has been postulated using a decoy molecule that causes the evicting amino acid doublet of the expressed SYT: SSX fusion gene to resemble the innocent SSX3 protein. The role of FOXM1 in synovial sarcoma needs experimental evaluation. However, inferring its effect on upregulating pluripotency genes and in particular activation of SOX2 in other tumors raises an important question: Does inhibition of FOXM1 vicariously targets SOX2 in synovial sarcoma? This postulate merits future research.

SOX2 also maintains the self-renewal of osteosarcoma tumor initiating cells [56]. In this malignancy SOX2 opposes the cellular differentiating effect of the Wnt pathway, which in turn can block SOX2 expression [59]. Interestingly constitutive Wnt/β-catenin signaling is aberrantly activated by SYT-SSX2 in synovial sarcoma [60]. In a general perspective SOX2 has been found to antagonize the Hippo pathway to maintain ‘stemness’ in cancer cells [58]. Evidence also exists that Yes-Associated Protein (YAP), the effector molecule which is inhibited by the Hippo signaling pathway cascade of cytoplasmic kinases, is increased in a subset of soft tissue sarcoma [31]. In these sarcomas i.e. fibrosarcoma, undifferentiated pleomorphic sarcoma and liposarcoma, YAP dependent expression of FOXM1 is necessary for cell proliferation and tumorigenesis.

Embryonic stem cells, embryonic germ cell and embryonal carcinoma (EC) cells are pluripotential cells. FOXM1 is required to maintain embryonal carcinoma cell pluripotency [30]. Embryonal carcinoma cells are derived from teratocarcinomas, with mice P19 EC cells derived from a genetically engineered murine teratocarcinoma. These cells can be induced to differentiate *in vitro* to any cell type of all three germ layers. There is an afore-described interesting parallel as sarcomas usually derive from mesodermal or ectodermal germ layers, in contrast to carcinomas, which arise from the endodermal germ layer. Some sarcomas such as angiosarcomas however have an endodermal origin establishing that soft tissue sarcomas as a group of heterogenous diseases with a possible common phylogenetic origin can arise from or developmentally transit all three germ layers. FOXM1 binds to and stimulates the Oct4 prompter in P19 EC cells. FOXM1 expression is repressed during retinoic acid induced differentiation of early stage P19 EC cells and FOXM1 knockdown in EC cells causes spontaneous differentiation to mesodermal derivatives such as adipose tissue or muscle [30]. Maintaining FOXM1 expression prevents down regulation of Nanog and Oct4 during P19 differentiation. When FOXM1 is overexpressed in human newborn fibroblasts or retinoic-acid differentiated cells, expression of Nanog, Sox2 and Oct4 is reinstated. Therefore the FOXM1 transcription factor maintains the pluripotency of P19 EC cells by up regulating pluripotency genes in this malignancy. Retinoic acid induced differentiation of human NT2/D1 embryonic carcinoma cells decreases expression of FOXM1 [61]. In a preclinical investigation this was found to be mediated by increased levels of miR-134 which binds to the FOXM1 3′UTR. In general miRNAs negatively regulate gene expression and are loaded onto RNA induced silencing complexes (RISC) with the miRNAs interacting with targeted messenger RNA. This interaction involves imperfect base pairing with microRNA response elements usually identified within the 3′UTR of the messenger RNA.

The general importance of FOXM1 upregulating pluripotency genes such as *SOX2, OCT4*, and *NANOG* is apparent beyond the specific cited examples. The histology of many sarcomas suggests a cellular pluripotency model of origin. Firstly, the World Health Organization (WHO) has a lineage of origin classification but some tumors such as hemangiopericytoma, fibrous histiocytoma or phosphaturic mesenchymal tumor do not have an identifiable lineage of origin. Sarcomas, which are poorly differentiated, are also increasingly difficult to assign a lineage of origin. The most common soft tissue sarcoma is undifferentiated pleomorphic sarcoma, a diagnosis of exclusion. Some sarcomas have histologic juxtaposition of malignant cells of different germ layer origin such as biphasic synovial sarcoma having both glandular cells (*endodermal origin*) and spindle cells (*mesenchymal origin*). Tumors with heterologous rhabdomyoblastic components also have close apposition of cells with different origins. This latter group has a sub-category of tumors with sarcomatous elements only (malignant mesenchymoma, dedifferentiated chondrosarcoma and dedifferentiated liposarcoma). Other subcategories with heterologous rhabdomyoblastic components include tumors with epithelial components (e.g. carcinosarcoma), malignant mixed Mullerian tumors, tumors with sex cord or stromal elements, and tumors of neuroectodermal derivation (e.g. medulloblastoma). A further observation supporting the pluripotent cell of origin in sarcomagenesis is the spatial heterogeneity of sarcoma types, often identified in body parts lacking a normal tissue of origin counterpart such as rhabdomyosarcomas occurring in areas lacking skeletal muscle. Finally in vicarious evidence of sarcomas originating from pluripotential or partly committed stem cells, sarcomas frequently do not arise from their intuitive benign precursor unlike colonic carcinomas arising from colonic polyps. Well-differentiated liposarcomas arise most commonly from deep soft tissues whereas lipomas usually occur in subcutaneous fat. Similarly uterine leiomyosarcomas do not arise from preceding uterine fibroids, monoclonal tumors derived from myometrium [62].

## FOXM1 AND EMBRYONIC PATHWAYS

Enhanced FOXM1 stability may arise from altered rate of FOXM1 production or degradation. Embryonic/stem cell pathways predominate in determining the cellular stoichiometry of FOXM1 levels. Wnt (effected by β-catenin) decreases the rate of FOXM1 degradation. Hedgehog signaling (effected by Gli) and Hippo signaling (effected by the YAP co-activator) up-regulate FOXM1[63]. Hypoxia inducible factor-1α, also upregulates FOXM1. Tumors in which FOXM1 is implicated have a temporal incidence consistent with this biology, such as anaplastic large cell lymphoma being the most common childhood form of non-Hodgkin's lymphoma. FOXM1 protein is stabilized in anaplastic large cell lymphoma by direct interaction with nucleophosmin (NPM). NPM is a fusion partner with ALK in 50%-75% of cases of anaplastic large cell lymphoma [64].

Frequently the interaction of FOXM1 with embryonal/stem cell preferential pathways is context dependent with variation occurring in different types of tumors. For example in endothelial cells FOXM1 has an unanticipated tumor suppressive function as it limits canonical Wnt signaling in lung epithelial cells [65]. Contrastingly, in gliomas FOXM1 promotes nuclear localization of β-catenin and expression of Wnt signaling pathway target genes [66]. Synovial sarcoma has a characteristic *SYT-SSX* translocation that increases Wnt pathway activity thereby imposing a differentiation block. Constitutive Wnt signaling activated by SYT-SSX2 has been identified in a *SYT-SSX2* translocated mouse model [60]. Genetic loss of β-catenin prevents synovial sarcoma formation.

FOXM1 is a direct transcription target of hedgehog signaling [67]. Aberrant hedgehog signaling occurs in basal cell carcinoma, medulloblastoma, and chondrosarcoma. In embryonal and fusion gene negative alveolar rhabdomyosarcoma activation of hedgehog signaling is associated with a poor prognosis [68]. Hedgehog signaling is also activated in desmoid tumors, a locally recurrent, non-metastasizing fibromatosis more common in familial adenomatous polyposis coli or Gardner's syndrome with strong nuclear accumulation of β-catenin. Enchondromas, which are benign cartilaginous tumors, have constitutively active Hedgehog signaling which blocks the normal differentiation of chondrocytes and promotes cellular proliferation. 1%-2% of cases of solitary enchondromas transform to chondrosarcoma, with 15%-30% of enchondromatosis such as Ollier disease or Mafucci's syndromes transforming to chondrosarcoma. Indian Hedgehog, one of the 3 hedgehog orthologous, is constitutively activated in primary central chondrosarcoma [69, 70].

The Hippo pathway is a kinase cascade that negatively regulates the transcriptional co-activator YAP, causing its' cytoplasmic retention, proteasomal destruction and nuclear exclusion. The upstream core-signaling axis of this pathway comprises the serine/threonine kinases MST1/2 followed by LATS1/2 [71]. Using information from The Cancer Genome Atlas Project, aberrant Hippo pathway signaling has been causally implicated in greater than 25% of sarcomas [31]. These comprise the histological subtypes fibrosarcoma, undifferentiated pleomorphic sarcoma, and liposarcoma. In this circumstance FOXM1 interacts directly with YAP (a transcription co-activator) *via* TEAD1 (the co-associating transcription factor) to alter regulation of pro-proliferative transcriptional targets. Inhibiting YAP decreases proliferation of sarcoma cells. YAP upregulates FOXM1. In murine models FOXM1 is required for sarcomagenesis. In a specific tumor type, dysregulation of Hippo signaling arises from a translocation fusion oncogene involving another FOX family member, FOXO1 in rhabdomyosarcoma. Rhabdomyosarcoma is the most common pediatric soft tissue sarcoma, accounting for 3% to 4% of all childhood cancers. Rhabdomyosarcoma can be either of alveolar or embryonal subtype. Alveolar rhabdomyosarcoma usually has a PAX3-FOXO1 translocation. This translocation oncogene permits bypass of cellular senescence by upregulating RASSF4, a Ras-associated domain family member. Enhanced RASSF4 expression inhibits MST1 to cause cell cycle progression and tumorigenesis [72]. YAP is upregulated in this circumstance.

## FOXM1 DIRECTED THERAPEUTICS

There are numerous promising preclinical and therapeutic strategies, applying mono- or combinatorial approaches, to target the oncogenic transcription factor FOXM1. These include RNA interference, thiazole antibiotics and proteasome inhibitors, the appreciation that standard anticancer drugs such as cisplatin suppress FOXM1, and targeting FOXM1 interaction with other proteins using a synthetic ARF related peptide or nucleophosmin. In a selected specific, targeting FOXM1 retards p53-null sarcoma and lymphoma [41].

Preclinical studies that ablate FOXM1 by RNA interference have demonstrated decreased cellular proliferation in numerous types of cancer including osteosarcoma [29]. Knockdown of FOXM1 causes increased cellular senescence of mAKT1 expressing osteosarcoma cells and deletion of FOXM1 can diminish cancer cell invasion, migration and angiogenesis [73]. The thiazole antibiotics, siomycin A and thiostrepton act as proteosomal inhibitors and inhibit FOXM1 transcriptional activity [74, 75]. Furthermore as FOXM1 has a positive auto regulation loop they also inhibit FOXM1 mRNA and protein expression [76]. In Ewing sarcoma thiostrepton has a dual inhibitory effect on FOXM1 and *EWS/FLI* mRNA expression [77]. Thiostrepton delays Ewing sarcoma xenograft growth in mice. FOXM1 is also a targeted for other proteasome inhibitors [74]. Bortezomib a proteasomal inhibitor already in clinical use has therapeutic efficacy previously attributed to suppression of NF-κB. Suppression of FOXM1 by proteasomal inhibition is an emerging alternative or complementary explanation [78]. In osteosarcoma and other cancer types a combination of bortezomib with thiostrepton induces profound apoptosis [79]. Several other anticancer drugs are also reported to repress FOXM1 including cisplatin, genistin (a chemo- preventive natural isoflavonoid in soya beans) and docetaxel [48].

The interaction of FOXM1 with other proteins can also be targeted. p19^ARF^ is one of the two gene transcripts and tumor suppressor products of the *CDKN2A* locus. It is a negative regulator of FOXM1. Amino acid residues 26-44 of p19ARF bind to the c-terminal of the transcriptional activator domain of FOXM1 to cause inhibition of FOXM1 transcriptional activity by sequestering FOXM1 to the nucleolus. A synthetic ARF peptide comprising (D-Arg)_9_-p19^ARF^26-44 has been created. This comprises nine D-Arg residues to allow cell penetration, and amino acid residues 26-44 of the ARF protein.

This ARF peptide induces apoptosis, decreases cellular proliferation and prevents pulmonary metastasis in hepatocellular carcinoma [80, 81]. In preclinical experiments this synthetic peptide suppressed FOXM1 induced colony growth of U2OS osteosarcoma cells and suppressed FOXM1 transcriptional activity [82, 83]. Nucleophosmin (a protein associated with the nucleolus) directly interacts with FOXM1 and alters its localization and levels in cancer cells [R14]. Both nucleophosmin and FOXM1 tend to co-localize in the nucleolus of cancer cells and RNAi- mediated nucleophosmin knockdown in cancer cells decreases levels of FOXM1. Small molecules, which interfere with the nucleophosmin-FOXM1 interaction, decrease levels of FOXM1 and may be effective in treating cancer.

Targeting transcription factors with small molecules is an important emerging field. BET (bromodomain and extra terminal domain) proteins are epigenetic reader proteins that cause highly transcription factor specific repression of gene expression [84].

The family of BET proteins comprising BRD2, BRD3, BRD4 and BRDT, contain 2 conserved tandem bromodomains, which have an epigenetic reading function recognizing acetylated lysine residues on histone tails [85]. I-BET and JQ1 are small molecule BET inhibitors, which mimic the acetyl moiety, block the acetyl lysine binding pocket of the bomodomains, and displace the BET proteins from chromatin [86, 87]. In many cancers the efficacy of BET inhibitors has been attributed to down regulation of the super enhancer dependent MYC transcript. However in contrast JQ1 markedly disrupts FOXM1 in sensitive ovarian cancer cells [88]. JQ1 is also efficacies in cell line and patient derived ovarian cancer xenograft models. Therefore epigenetic targeting of FOXM1 with small molecules is a potential therapeutic approach. JQ1 induces antiproliferative activity and apoptosis in osteosarcoma cells by suppressing the transcription factor FOSL1 [89]. This is mediated by displacing BRD4 from its locus. A synergistic increase in osteosarcoma cell sensitivity was observed with combined treatment of JQ1 with CDK inhibitors. The role of FOXM1 in this context requires future research.

FOXM1 can mediate drug sensitivity or resistance, and in breast cancer FOXM1 overexpression confers resistance to trastuzumab and paclitaxel. Stathmin a tubulin destabilizing protein that confers resistance to paclitaxel is a direct transcription target of FOXM1. In contra-distinction paclitaxel mediates it's anti-cancer activity by stabilizing the microtubule assembly. Attenuation of FOXM1 expression by RNA interference or alternative reading frame derived peptide inhibitors increased the therapeutic sensitivity of breast cancer to paclitaxel [90]. In pediatric B-acute lymphoblastic leukemia FOXM1 is overexpressed and in cell lines inhibition of FOXM1 with thiostrepton enhanced cytotoxicity to commonly used chemotherapeutic agents (dexamethasone, asparaginase, daunorubicin, vincristine and Ara-C) [91].

FOXM1 is overexpressed in many types of cancer and is usually absent in terminally differentiated cells. This differential expression renders it an attractive therapeutic target. It is likely that sarcoma subtypes with a high differentiation score are more likely to be sensitive to therapeutic inhibition of FOXM1. The commonly used three tiered FNCLCC (Fédération Nationale des Centres de Lutte Contre le Cancer) grading system of tumor differentiation, mitotic count, tumor necrosis and histologic grade are used as criteria to categorize sarcomas. The highest tumor differentiation score of 3 is assigned to the following sarcoma subtypes: round cell liposarcoma, pleomorphic liposarcoma, dedifferentiated liposarcoma, undifferentiated pleomorphic sarcoma, undifferentiated spindle cell sarcoma, poorly differentiated/pleomorphic/epithelioid leiomyosarcoma, biphasic/monophasic synovial sarcoma, pleomorphic rhabdomyosarcoma, mesenchymal chondrosarcoma, extraskeletal osteosarcoma, Ewing sarcoma/PNET, malignant rhabdoid tumors and some MFH variants. It is noteworthy that several sarcoma subtypes are exempted as grading of them is not recommended [92].

Nutlins are small molecule imidazoline compounds that selectively and potently inhibit MDM2 thereby stabilizing and activating p53 [93]. MDM2 is an E3 ligase, which binds p53, blocks the p53 transactivation domain and causes p53 degradation [94]. Nutlins suppress levels of FoxM1 in cell line studies. Inhibition of MDM2 by nutlins in liposarcoma activates the p53 pathway with decreased cellular proliferation in MDM2-amplified liposarcoma [95]. In one proof of principle study of 20 patients with well differentiated or dedifferentiated liposarcoma (18 TP53 wild-type, 2 TP53 missense mutant) treated with the nutlin RG7112, one patient had a complete response with fourteen patients experiencing stable disease. In a separate study involving melanoma, AMG 232 a small molecule that inhibits the MDM2-p53 interaction was combined with radiation treatment in cell lines and xenograft models [96]. Treatment with radiation or AMG 232 alone caused minor reductions in FOXM1 levels, whereas the combination treatment caused significant reductions in FOXM1 levels. This was detected by immunohistochemistry of treated A375 melanoma cells.

CDK4 is amplified in over 90% of well-differentiated and de-differentiated liposarcomas. Palbociclib (a CDK4/6 inhibitor) is associated with a favorable progression free rate in RB expressing, CDK4 amplified well-differentiated and de-differentiated liposarcomas that experienced prior progression on systemic treatment [97]. FOXM1 is elevated in de-differentiated liposarcoma and as FOXM1 is activated by progressive phosphorylation the relationship between palbociclib, CDK4 and FOXM1 in liposarcoma merits future research [31].

## CONCLUSIONS

The central role of FOXM1 in the cell cycle, and it's recurring theme of importance in stem cell preferentially activated developmental pathways suggests that targeting FOXM1 is a potential advance in treating sarcoma. This review infers that certain sarcoma subtypes such as Ewing sarcoma and osteosarcoma may be particularly ‘druggable’ using FOXM1 inhibition. The accruing preclinical evidence presents a substantiate rationale for clinical trials in the future.
